# On the Biomechanical Performances of Duplex Stainless Steel Graded Scaffolds Produced by Laser Powder Bed Fusion for Tissue Engineering Applications

**DOI:** 10.3390/jfb14100489

**Published:** 2023-09-22

**Authors:** Maria Laura Gatto, Giorgia Cerqueni, Riccardo Groppo, Emanuele Tognoli, Alberto Santoni, Marcello Cabibbo, Monica Mattioli-Belmonte, Paolo Mengucci

**Affiliations:** 1 Department of Industrial Engineering and Mathematical Sciences (DIISM), Marche Polytechnic University, Via Brecce Bianche 12, 60131 Ancona, Italy; m.l.gatto@univpm.it (M.L.G.); a.santoni@pm.univpm.it (A.S.); m.cabibbo@univpm.it (M.C.); 2 Department DISCLIMO & UdR INSTM, Marche Polytechnic University, Via Tronto 10/a, 60126 Ancona, Italy; g.cerqueni@univpm.it (G.C.); m.mattioli@univpm.it (M.M.-B.); 3 3D4MEC S.r.l., Via Porrettana 48, 40037 Sasso Marconi, Italy; riccardo.groppo@3d4mec.com; 4 Department of Engineering “Enzo Ferrari”, University of Modena and Reggio Emilia, Via Vivarelli 10, 41125 Modena, Italy; emanuele.tognoli@unimore.it; 5 Department SIMAU & UdR INSTM, Università Politecnica delle Marche, Via Brecce Bianche 12, 60131 Ancona, Italy

**Keywords:** duplex stainless steel, laser powder bed fusion, tissue engineering, MG63 cells, mechanical behavior

## Abstract

This experimental study aims to extend the know-how on biomechanical performances of duplex stainless steel (DSS) for tissue engineering applications to a graded lattice geometry scaffold based on the F53 DSS (UNS S32750 according to ASTM A182) produced by laser powder bed fusion (LPBF). The same dense-out graded geometry based on rhombic dodecahedral elementary unit cells investigated in previous work on 316L stainless steel (SS) was adopted here for the manufacturing of the F53 DSS scaffold (SF53). Microstructural characterization and mechanical and biological tests were carried out on the SF53 scaffold, using the in vitro behavior of the 316L stainless steel scaffold (S316L) as a control. Results show that microstructure developed as a consequence of different volume energy density (VED) values is mainly responsible for the different mechanical behaviors of SF53 and S316L, both fabricated using the same LPBF manufacturing system. Specifically, the ultimate compressive strength (σUC) and elastic moduli (E) of SF53 are three times and seven times higher than S316L, respectively. Moreover, preliminary biological tests evidenced better cell viability in SF53 than in S316L already after seven days of culture, suggesting SF53 with dense-out graded geometry as a viable alternative to 316L SS for bone tissue engineering applications.

## 1. Introduction

The most common austenitic stainless steel (SS) used for manufacturing osteosynthesis devices is 316L [1], although applications are confined to temporary bone healing, such as screws and fixations [2], due to sensitivity to crevice corrosion. Prolonged contact with body fluids increases the likelihood of 316L SS localized corrosion [3], thus promoting the release of metallic ions into the adjacent tissues and the degradation of the implant. In this regard, the release of nickel (Ni) in concentrations exceeding the admissible level causes allergy, carcinogenicity, cytotoxicity and genotoxicity [3]. As per the in-use mechanical behavior, 316L SS exhibits inadequate fatigue resistance in several orthopedic devices subjected to exceptionally high stresses, such as Harrington rods for treating scoliosis and sliding-compression plate-screw systems used in fracture fixation procedures [4]. Consequently, numerous experimental studies in the literature have focused on providing an alternative to the austenitic SS employed in the biomedical field, using duplex stainless steels (DSS). DSS shows a double-phase structure containing approximately equal amounts of austenite (γ-Fe) and ferrite (α-Fe), resulting in a suitable combination of mechanical properties and resistance to corrosion [5].

Cigada et al. [6] found higher fatigue limits of the 25Cr-7Ni-4Mo-0.28N DSS (2507 alloy) relative to the 18Cr-14Ni-2.5Mo austenitic SS (ASTM F138), investigated in the same cold-worked conditions. Furthermore, Gregorutti et al. [3] compared the mechanical response of the 2507 DSS to the 18Cr-12.5Ni-2.5Mo austenitic SS (ASTM F745), showing that the yield strength (YS) and ultimate tensile strength (UTS) of tensile tested samples processed by investment casting are almost twice as large in DSS [3]. Such enhancement of mechanical strength in DSS was attributed to the smaller grain size typical of DSS and to solid solution hardening, promoted by the higher content of substitutional Cr and Mo atoms as well as of interstitial N atoms [3]. Moreover, elongation tests carried out under the same conditions provided 20% for DSS and up to 30% for the austenitic SS. The higher ductility of the SS was attributed to its fully austenitic structure, while the presence of ferrite, in addition to austenite in DSS, reduces ductility and increases mechanical strength [3].

The in vitro corrosion behavior of DSS has been deeply investigated in the literature by degradation tests in different artificial physiological solutions, resulting in lower susceptibility to localized corrosion relative to austenitic SS [3,4,6,7,8,9,10,11]. Several studies also focused on the improvement of surface corrosion behavior following surface treatment with electrical discharge machining (EDM) [7] and the coating of a hydroxyapatite (Hap) and hydroxyapatite/titania (Hap/TiO_2_) nanocomposite [5] using an electrophoretic deposition (EPD) process [12].

In vivo investigations were conducted on sheep and rabbits to assess the biocompatibility and the in vivo localized corrosion resistance of cold-worked 2507 DSS, compared to AISI 304L and ASTM F138 austenitic SS [6]. After implant, examination of femur sections from rabbits and sheep demonstrated a bone formation in proximity to the metal implants, devoid of any intervening connective tissue. However, the results of corrosion tests indicated the occurrence of pitting corrosion on the AISI 304L, crevice corrosion on the ASTM F138, while no evidence of corrosion was observed on the 2507 DSS [6]. Based on the in vivo results obtained, a preliminary clinical investigation was carried out on patients by the implantation of two or more Ender-type endomedullary nails in 2507 DSS and ASTM F138, inserted in the femoral neck for six months [6]. A significant portion of Ender nails produced in ASTM F138 showed severe crevice corrosion, which originated from the interface region between the nails. By contrast, the application of 2507 DSS resulted in minor fretting and the absence of crevice corrosion [6]. Moreover, Cigada et al. [13] developed a device consisting of two metallic disks sandwiched between two PTFE disks, for evaluating the in vivo resistance to localized corrosion of ASTM F138 and 2507 DSS. This anti-corrosion device was surgically inserted into the trochanteric region of the femurs of rabbits. After 12 months of device implant, the occurrence of pitting corrosion was noticed on the AISI 304 austenitic SS. Additionally, crevice corrosion was detected on the ASTM F138 austenitic SS; however, no signs of corrosion were observed on the 2507 DSS. The main corrosion occurred at the interface between metal and PTFE [13]. Therefore, in vivo results in the literature indicate that the 2507 DSS is not susceptible to crevice corrosion within the human body, at least under static conditions, and can be considered a good biocompatible material.

Thence, DSS is a viable alternative to austenitic stainless steels for bone tissue engineering applications, due to its crevice resistance in chloride media, high mechanical strength and biocompatibility [3]. However, DSS use in the biomedical field must still be validated, mainly due to the limited understanding of DSS behavior when exposed to magnetic fields in terms of device heating or movement that can be injurious to the human body [3].

In bone tissue regeneration, a focal point for scaffold fabrication is elementary unit cell geometry, which influences the mechanical and biological performances of the designed structure [14]. Functional grading of unit cells is a promising approach in addressing the biomechanical requirements of bone, since it allows to program the deformation behavior of the scaffold by controlling the local relative density of unit cells. Thus, graded reticular structures enable the design of implants with local stiffness matching that of the target bone [15].

Additive manufacturing (AM) exhibited remarkable advantages for the fabrication of metal parts of customized geometry, and laser powder bed fusion (LPBF) technology has been widely investigated to produce DSS. Fine control of the LPBF processing parameters enables a comprehensive analysis of the DSS microstructural evolution as well as formation quality, which are critical points for designing the performance of biomedical devices, including mechanical properties and resistance to corrosion. Nevertheless, phenomena involved in the LPBF production process, such as laser-powder interaction drawbacks and extremely high solidification rates, make the fabrication of devices with high surface quality and a predictable microstructure still quite challenging [16].

To date, there is a lack of studies focused on the behavior of DSS fabricated by AM for tissue engineering applications. Furthermore, no studies have addressed the production of DSS scaffolds for tissue engineering applications with graded lattice geometries, although graded structures have been shown to be suitable for enhancing tissue ingrowth and for tailoring the biomechanical response of scaffolds [17,18,19]. In a previous paper we reported on the structural, mechanical and biological performances of additively manufactured graded scaffolds based on the 316L austenitic stainless steel in order to investigate their biomechanical response in perspective of bone tissue regeneration [15]. Taking as a reference the results obtained on the 316L scaffolds [15], in this paper we report on the biomechanical response of additively manufactured graded scaffolds based on the F53 DSS. The aim of this experimental work is to investigate the biomechanical properties of DSS graded scaffolds produced by LPBF and compare such properties to the in vitro behavior of the 316L scaffolds studied in our previous work [15]. Following the results of our previous work on S316L [15], dense-out (DO) graded geometry based on the rhombic dodecahedral (RD) elementary unit cell was adopted here for the manufacturing of the DSS scaffolds. After production, the DSS scaffolds were submitted to microstructural, mechanical and biological characterization, and their biomechanical response was compared to the behavior of the S316L scaffolds produced and investigated under similar conditions in a previous paper [15].

## 2. Materials and Methods

### 2.1. Scaffold Design

**Figure 1:**
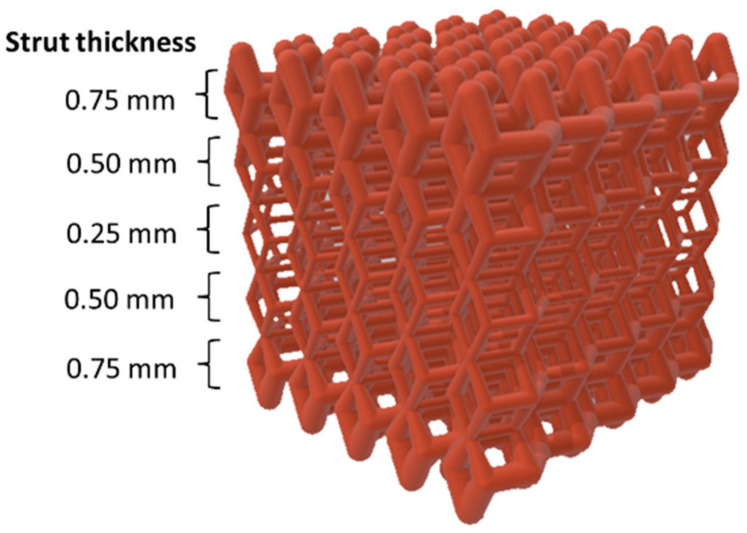
Scheme of dense-out (DO) scaffold geometry. Values of strut thickness (unit: mm) for cells arranged along the scaffold layers are reported next to the scaffold.

The same dense-out (DO) graded lattice geometry adopted in our previous work on 316L SS [15] was used in this experimental work (Figure 1). Cubic scaffolds with 10 mm sides with a total scaffold volume Vs. = 1000 mm^3^ were obtained by repeating in space a rhombic dodecahedral (RD) elementary unit cell. The graded structure was achieved by varying the strut size of the elementary unit cell layer by layer along the scaffold building direction. Values of strut thickness were in the range of 0.25–0.75 mm, with a step size of 0.25 mm. Strut size increased from core to edge, and the geometry was built in specular symmetry from the central horizontal axis. Thus, the scaffold was composed of a total of five layers (Figure 1). The values from STL files of the total volume of material (V_mSTL_) and porosity (P_STL_) were respectively V_mSTL_ = 280 mm^3^ and P_STL_ = 72%.

### 2.2. Scaffold Manufacturing

The commercial raw powder MARS F53 (UNS S32750, according to ASTM A182) produced by vacuum inert gas atomization was provided by Mimete Srl (Milano, Italy). As from the manufacturer data sheet, powder particles are spherical with a size in the range of 15–45 µm, and their nominal composition is reported in Table 1.

Scaffolds with DO geometry were produced starting from the F53 virgin new powder by using the laser powder bed fusion (LPBF) technology in a 3D4steel manufacturing system (3D4MEC Srl, Sasso Marconi, Italy). The production system was equipped with a 300 W Yb-fiber laser operating in a nitrogen atmosphere. Optimization of printing parameters was performed producing cubic samples with 10 mm sides with variable values of laser power and scanning distance until a relative density of 98.6% was obtained. The optimized printing parameters for scaffold manufacturing are reported in Table 2. The volume energy density (VED) corresponding to the optimized parameters is E_v_ = 60 J/mm^3^. The scanning strategy for scaffold production involves an initial consolidation of the heart of the column and then a scan that delimits its contours. Scaffolds were studied in the as-produced condition, without any post-processing treatment. The size accuracy of the manufactured structure has a deviation from the designed dimension estimated as +/− 0.1 mm.

To investigate possible compositional variations of the manufactured scaffolds with respect to the virgin new powder and to evidence possible dealloying phenomena attributable to vaporization of most volatile elements during the laser melting process, the structure and composition of the residual powder inside the chamber after scaffold production were considered. Therefore, from here on the investigated samples are distinguished as: (a) PF53—virgin new powder; (b) PF53R—residual powder in the chamber after scaffold production; (c) SF53—scaffold produced from the virgin new powder.

### 2.3. Structural Characterization

The morphology of PF53 and PF53R powders was observed using a Zeiss Supra 40 field emission scanning electron microscope (FESEM) (Carl Zeiss, Oberkochen, Germany), while the scaffold surface, inner structure, mechanical deformation and cell adhesion were investigated with a Tescan Vega 3 scanning electron microscope (SEM) (Tescan, Brno, Czech Republic). To observe the inner structure of the SF53 sample, the scaffold was cut by a diamond saw at half height, which is about 5 mm from the scaffold top surface.

A Bruker Z200 energy dispersive microanalysis (EDS) (Billerica, MA, USA) was employed to analyze the chemical composition of samples. Results were obtained by averaging the data taken from five different areas of the sample, observed at the same magnification (300×).

Information on the crystallographic structure of samples was achieved by X-ray diffraction (XRD), using a Bruker D8 Advance diffractometer (Bruker, Karlsruhe, Germany) with Cu-Kα radiation, operating at V = 40 kV and I = 40 mA, in the angular range 2θ = 30–90°. Pattern analysis was performed using the DIFFRAC.EVA software package (Version 4.3.0.1, Bruker) and peak indexing was carried out using the search/match facility using the PDF 2 database of the international center for diffraction data (ICDD). XRD peaks shape analysis including estimation of the lattice parameters of the α-Fe (ICDD 6-696) and γ-Fe (ICDD 33-397) phases calculated by nonlinear curve fitting analysis was conducted using the OriginPro 2023 software (Origin Pro, Version 2023. OriginLab Corporation, Northampton, MA, USA). The crystallite size was calculated using the Scherrer formula from the most intense peaks of phases, which are α-Fe (110) for the body centered cubic (bcc) phase and γ-Fe (111) for the face centered cubic (fcc) phase. Rietveld refinement was performed using the MAUD software, following [20] allowed estimating the amount of phases in all the investigated samples.

Metallographic analysis was carried out on the SF53 scaffold on the x–y plane, perpendicular to the build direction (*z*-axis), and on the y–z plane, along the build direction, by a Leica DMi8 optical microscope (Leica Microsystems, Wetzlar, Germany). Samples were prepared by etching in an electrolytic solution of 10 g oxalic acid in 100 mL water at 6 V for 45 s.

### 2.4. Mechanical Tests

Compression tests were performed on five samples of the SF53 scaffolds using an INSTRON 5567 machine (Instron, Norwood, MA, USA), with a 30 kN load cell at 0.5 mm/min speed. The test was interrupted at 30% of scaffold compression extension. Compression data were plotted as load–strain curves. The elastic modulus E was calculated using regression linear statistics in the load range of 4 to 8 kN of the stress–strain curves. Ultimate compressive strength (σ_UC_) was determined out of the stress–strain curves.

### 2.5. Biological Tests

Biological tests were performed on the SF53 scaffold, using the S316L scaffold as a control.

MG-63 human osteoblast-like cells (CRL-1427, American Type Culture Collection (ATCC), Manassas, VA, USA) were maintained in Dulbecco Modified Eagle’s Medium (H-DMEM, D6429, Sigma-Aldrich, Burlington, MA, USA) with 1% penicillin–streptomycin (15140122, Thermo Fisher Scientific, Waltham, MA, USA) and 10% FBS (35-079-CV, Corning, New York, NY, USA), in a humidified incubator (Eppendorf, Hamburg, Germany) at 37 °C with 5% CO_2_. For passaging, trypsin/EDTA (trypsin 0.05%—EDTA 0.02% in PBS, Sigma-Aldrich, Burlington, MA, USA) was used. The medium was refreshed every three days.

The SF53 and S316L scaffolds were autoclaved at 120 °C for 20 min and then UV-exposed for 30 min on the top and bottom surfaces along the build direction. After sterilization, samples were conditioned overnight with H-DMEM with 10% FBS and 1% penicillin/streptomycin.

The indirect cytotoxicity of scaffolds was evaluated according to ISO 10993-12 [21] by using the material-conditioned media (CM). MG63 were seeded at a density of 2 × 10^4^ cells per well, in 96 wells/plates. After 24 h from seeding, the medium was replaced with S316L and SF53 CM or their 1:2 dilution. Then, an MTT assay was performed after 24 h and 72 h and H-DMEM was used as a control.

After the materials’ preconditioning, direct cytocompatibility tests were performed to evaluate cell–materials interaction. Each scaffold was placed in a well of 12 wells/plates and seeded with 8 × 10^4^ MG63 cells. Scaffolds were incubated at 37 °C with 5% CO_2_ and the medium was replaced every two days. After 24 h, 72 h and 7 d from seeding, MTT assays and SEM observations were performed on the SF53 and S316L scaffolds.

Following [15], live and metabolically active MG63 cells were assayed by MTT (3-dimethylthiazol-2,5-diiphenyltetrazolium bromide, Sigma-Aldrich, M5655, Sigma-Aldrich, Burlington, MA, USA) according to the manufacturer’s instructions. Briefly, the MTT stock solution (5 mg/mL) was diluted 1:10 in cell culture medium and incubated at 37 °C for 3 h. After incubation, the medium was removed and 100 µL of DMSO were added to each well to dissolve the dark blue formazan crystals. Then, the absorbance was quantified by spectrophotometry (MultiskanGo, Thermo Scientific, Pittsburgh, PA, USA), monitoring the absorbance at 570 nm with a reference wavelength at 650 nm.

For ultrastructural morphology, cells were fixed in 2% glutaraldehyde (MERCK, 4239, Rahway, NJ, USA) in 0.1 M sodium cacodylate buffer (C-0250, Sigma-Aldrich, Burlington, MA, USA), followed by washes in 7% sucrose in 0.1 M cacodylate buffer. Post-fixation was carried out in 1% osmium tetroxide (Electron Microscopy Sciences, 12310, Hatfield, PA, USA) in 0.1 M sodium cacodylate buffer. Complete dehydration was achieved in graded alcohol series (from 25% to 100%) and Critical Point Dry was performed with hexamethyldisilane (HMDS, 440191, Sigma-Aldrich, Burlington, MA, USA). Scaffolds were then observed by SEM.

Three separate biological experiments were performed, and each test was conducted in triplicate. Two-way ANOVA analysis followed by Sidak’s multiple comparison test was applied to determine the differences between the experimental groups. Differences were considered statistically significant when *p* < 0.05.

## 3. Results

### 3.1. Structural Characterization

**Figure 2:**
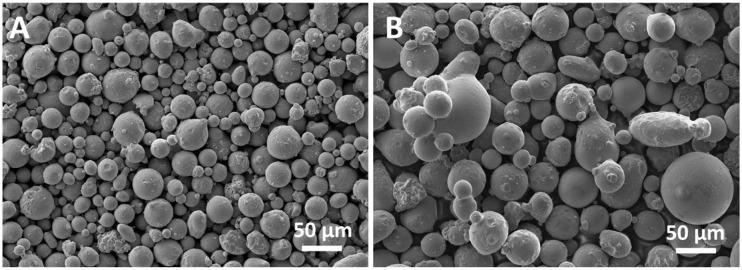
FESEM images of powders: (**A**) PF53 virgin new powder and (**B**) PF53R residual powder in the chamber after scaffold production.

FESEM images of the virgin new powder (PF53) and residual powder in the chamber after scaffold production (PF53R) are shown in Figure 2. PF53 mainly exhibited spherically shaped particles with a size in the range of 5 ÷ 50 µm and some satellite particles partially melted on the surface of the larger ones (Figure 2A). On the other hand, the PF53R powder showed more irregular particles resulting from the aggregation of melted or partially melted particles (Figure 2B). The size of particles in the PF53R powder ranged from 5 to 130 µm.

**Figure 3:**
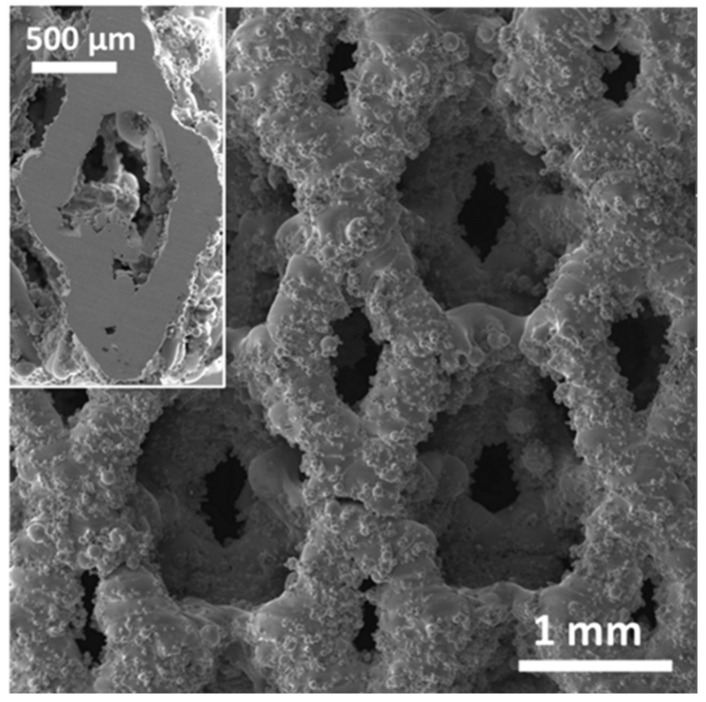
SEM image of the SF53 scaffold top surface. Inset shows the SF53 inner structure after cutting the scaffold at half height (about 5 mm from the scaffold top surface).

The surface morphology of the SF53 scaffold is shown in Figure 3, along with a detail of the inner structure after cutting the scaffold at half height (inset in Figure 3). Powder particles remained partially melted on the struts, while pores were free of residual powder (Figure 3). The size of particles of the residual powder on the scaffold surface was smaller than the size of the particles of the virgin new powder, due to their partial melting during the LPBF process. The SEM image in the inset of Figure 3 reveals the presence of closed micro-pores within the struts.

As shown in Table 3, within the statistical uncertainties, a decrease of the Mn content in the SF53 scaffold is evident along with a contemporary increase of the same element in the residual PF53R powder, while the composition of the PF53 virgin new powder is compatible with the nominal one (Table 1).

**Figure 4:**
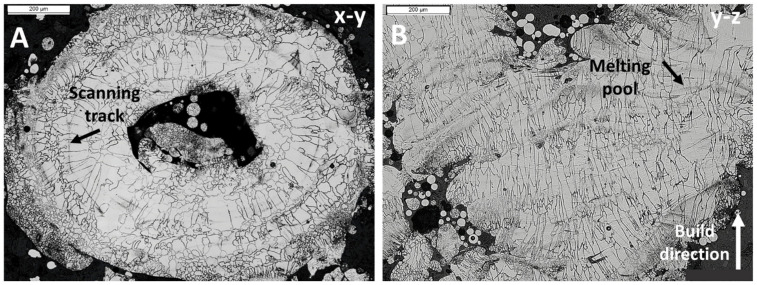
Optical micrographs of SF53 microstructure: (**A**) x–y plane—scanning track of laser is indicated by a black arrow; (**B**) y–z plane—laser melting pool is marked with a black arrow, while the build direction is marked with a white one.

Metallography observations of the F53 scaffold (SF53) on the x–y and y–z planes are reported in Figure 4. Figure 4A shows a cross-section of the elementary cell on the x–y plane, while Figure 4B shows a cross-section on the y–z plane. On the x–y plane (Figure 4A), grains grew epitaxially at the interface with the macro-pores and radially elongated along the solidification direction from the inside out. Furthermore, the scanning tracks of the laser are recognizable. On the other hand, on the y–z plane (Figure 4B), the grains are oriented along the built direction and laser melting pools are visible.

**Figure 5:**
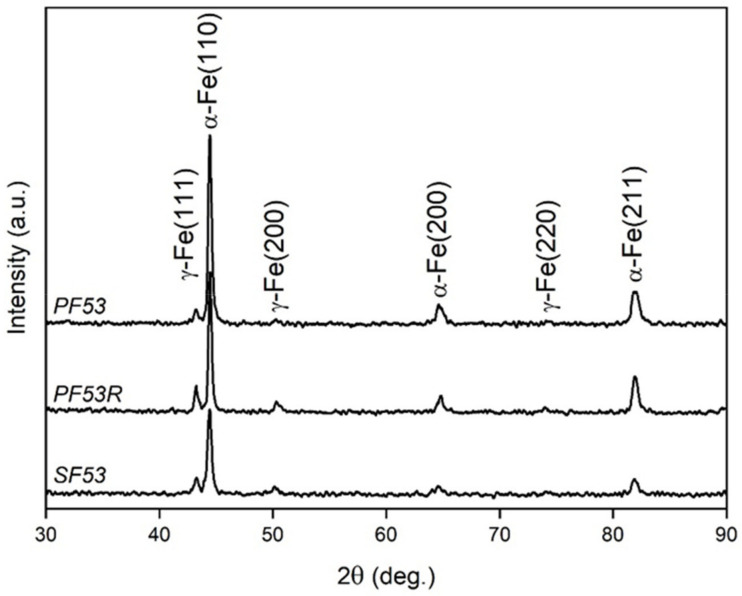
XRD patterns of PF53, PF53R and SF53 obtained with identical instrumental conditions.

XRD patterns of all the investigated samples are reported in Figure 5. The patterns in Figure 5 are vertically translated to ease comparison. Peaks revealed the presence of the γ-Fe (austenite) face-centered cubic (fcc) phase (ICDD 33-397) with nominal lattice parameter a = 0.35911 nm and the α-Fe (ferrite) body-centered cubic (bcc) phase (ICDD 6-696) with nominal lattice parameter a = 0.28664 nm.

The results obtained from peak shape analysis and Rietveld refinement are reported in Table 4.

The results in Table 4 clearly show different weight fractions of α-Fe and γ-Fe phases in the scaffold (SF53) as well as in the residual powder (PF53R) relative to the virgin new powder (PF53), due to melting and thermal cycling during manufacturing. Furthermore, while the crystallite size of the γ-Fe phase is almost the same in the powders (PF53 and PF53R), it decreases in the scaffold (SF53), where melting and rapid solidification occur during the LPBF process. By contrast, the α-Fe phase is more sensitive to heat treatments, showing an increase in crystallite size in the PF53R residual powder, which undergoes thermal cycling, and a significant size reduction after the melting and rapid cooling process due to the laser action (SF53).

### 3.2. Mechanical Tests

**Figure 6:**
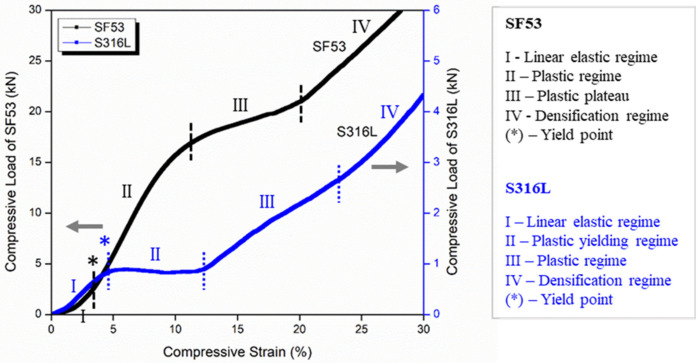
Mechanical response under compression of SF53 and S316L scaffolds with the same dense-out graded lattice geometry. Curves are plotted in different full-scale values and arrows indicate the reference scales for SF53 and S316L.

The load–strain compressive curve of the SF53 scaffold is plotted in Figure 6 along with the curve of S316L from our previous work [15], for comparison. The different full-scale values of the compressive load for the two materials are worth noting.

Figure 6 highlights the different mechanical behavior of the SF53 and S316L scaffolds under compressive load. In the compressive curve of S316L, four different regimes can be recognized: (I) elastic; (II) plastic yielding; (III) plastic; and (IV) densification. On the other hand, the initial linear behavior of SF53 (I) is followed by the plastic regime (II), until reaching a plateau around 20 kN of load (III). After this, the compression curve of SF53 shows a densification regime (IV).

The ultimate compressive strength (σ_UC_) and elastic modulus (E) for SF53 and S316L, estimated from compression test, are reported in Table 5.

**Figure 7:**
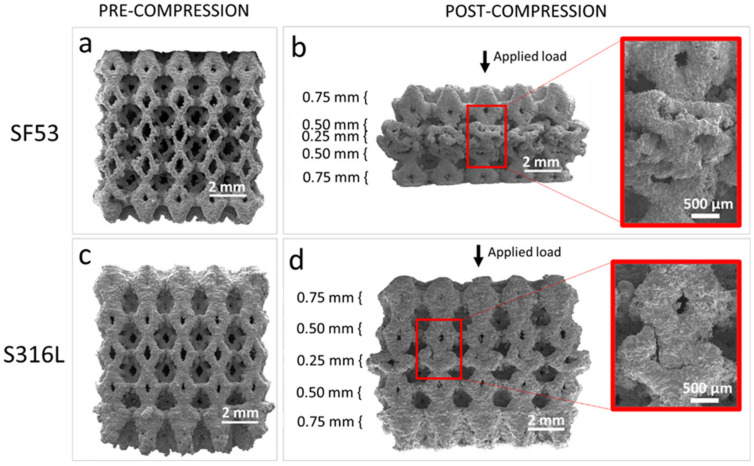
SEM images of SF53 graded lattice scaffold before (**a**) and after (**b**) compression test. Micrographs of S316L before (**c**) and after (**d**) compression are reported for comparison. The direction of the applied load is shown by vertical arrows. The dimensions of the unit cell’s strut size are reported for each layer. Inset shows the compaction of cells with thinner struts at higher magnification.

The failure mechanisms of the SF53 and S316L scaffolds were observed by SEM (Figure 7). SEM micrographs of SF53 before (Figure 7a) and after (Figure 7b) mechanical compression tests clearly shown that the plastic deformation of the scaffold first involved the elementary cells with the smallest strut size (0.25 mm). SEM observations of S316L before (Figure 7c) and after (Figure 7d) compressive test are reported in Figure 7 for comparison.

### 3.3. Biological Tests

**Figure 8:**
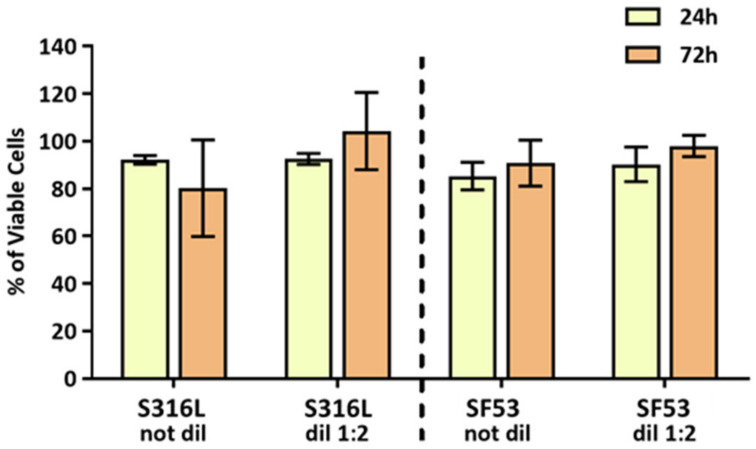
Viability of MG63 cells incubated for 24 h and 72 h with non-diluted and diluted 1:2 CMs derived from S316L and SF53 scaffolds. Data are expressed as % of cells cultivated with normal medium.

The results of indirect cytotoxicity assessment are shown in Figure 8. The influence of conditioned mediums (CMs) derived from the S316L and SF53 scaffolds on MG63 viability was investigated by MTT assay after 24 and 72 h of incubation (Figure 8A). No significant differences were detected between non-diluted and diluted CMs, as well as between SF53 and S316L.

**Figure 9:**
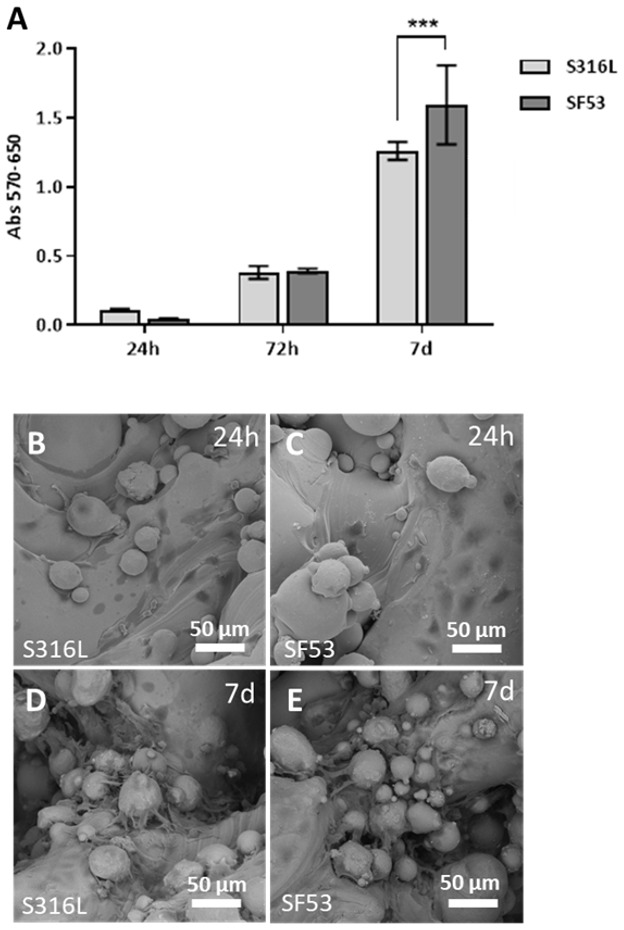
Viability of MG63 cells seeded on the S316L and SF53 scaffolds: (**A**) MTT assay results at 24 h and 7 days. Data are expressed as Absorbance 570–650; (**B**–**E**) cell morphology on S316L and SF53 after 24 h (**B** and **C**, respectively) and after 7 days (**D** and **E**, respectively; *** *p* < 0.001).

Cells–materials direct interactions were investigated using MTT assays (Figure 9A) and the ultrastructural morphology was observed by SEM (Figure 9B–E). The viability of MG63 increased in a time-dependent manner on both scaffolds; however, after seven days of culture, SF53 showed a significant (*p* < 0.001) higher increase of viable cells than S316L (Figure 9B). MG63 cells adhered on both stainless steels already at 24 h after seeding, displaying an elongated and spread morphology (Figure 9B,C). In addition to the surface strut, semi-melted particles offer anchorages to cells for adhesion. After seven days of culture, the S316L and SF53 scaffolds were completely covered by MG63 human osteosarcoma cells (Figure 9D,E).

## 4. Discussion

In our previous study [15], two different graded lattice structures fabricated in 316L stainless steel (SS) by laser powder bed fusion (LPBF) were investigated, aiming to design scaffolds with improved biomechanical performances for bone tissue regeneration. Dense-in (DI) and dense-out (DO) scaffolds were obtained, varying the strut size of a rhombic dodecahedral elementary unit cell layer-by-layer, along the build direction. The strut size decreased in DI and increased in DO from the core to the edge of the scaffold, with specular symmetry relative to the central layer. The combined control of dense-out grading strategy, printing parameters and elementary unit cell geometry allowed implementing scaffolds with improved biomechanical performances. Therefore, taking as a reference the results obtained on the 316L scaffolds [15], in this paper we report on the biomechanical response of a dense-out graded scaffold (Figure 1) based on the F53 duplex stainless steel (DSS) and fabricated by LPBF.

According to the content of Cr, Mo and N, stainless steels show resistance to pitting corrosion, empirically quantified by the pitting resistance equivalent number (PREN), defined as PREN = Cr% + 3.3Mo% + 16N%. A higher PREN value is associated with a lower tendency to pitting corrosion, thus resulting in a useful parameter for comparing and ranking the grades of duplex stainless steels. DSSs with a PREN higher than 40 were reported to be extremely resistant to pitting corrosion and are designated as super-DSS [22]. The F53 alloy used in this study shows a PREN ranging from 38 to 48, based on the chemical composition provided by the manufacturer (Table 1), thus resulting in a super duplex stainless steel.

Experimental chemical composition of F53 virgin new powder (PF53) is within the range of nominal composition (Table 3). However, the F53 scaffold fabricated with PF53 (SF53) shows a decrease in the amount of Cr and Mn, due to their preferential vaporization during the remelting process, as shown by Li et al. [23], as the process of remelting occurs from zero to six times in LPBF 22Cr-6Ni built with a 90° rotation strategy. During the LPBF process, in the keyhole region of the melting pool, the temperature exceeds the boiling point of metal, and the vapor pressure inside the keyhole is higher than the ambient pressure. The excess pressure provides a driving force for the vapor to move away from the surface. Therefore, the convective flux of the vaporized elements contributes to the vaporization loss of elements [24]. Specifically, the pressure-driven vaporization loss of elements occurs as follows: (I) transportation of vaporization elements from the scaffold to the surface of the melt pool; (II) vaporization of elements at the liquid–vapor interface; and (III) transportation of the vaporized elements into the surrounding atmosphere [24]. By such a mechanism, the redeposition of vaporized Mn on the powder bed during manufacturing of SF53 occurs, as demonstrated by the increase in the Mn amount shown by the residual powder in the chamber after scaffold production.

The detailed examination of the residual powder in the chamber after scaffold production (PF53R) finds reason in the possibility of recycling powder after the LPBF process. However, recycled powder could undergo degradation, depending on the alloy chemical composition, the processing parameters employed and the building environment [25]. For instance, the interaction between the laser and powder particles during the LPBF process leads to morphology modification of reused powder, which includes powder clusters, multiple satellites and shape-deformed particles [25], as shown for the PF53R in Figure 2B. Moreover, reused powder could present altered phases, likely due to the large thermal gradient in the LPBF process promoting solid-state phase transformation [25].

In this work, a volumetric energy density (VED) value of 60 J/mm^3^, resulting from the optimized printing parameters in Table 2, allowed producing bulk samples of the F53 alloy with a relative density of 98.6% and SF53 scaffold containing 80 wt.% of the ferritic phase (Table 4), in agreement with the literature results on microstructure evolution. Mulhi et al. [26] in their study of the UNS S32750 DSS (2507 alloy) produced by LPBF, correlated the VED to the relative density and microstructure of samples. In particular, they found an increase of the ferrite fraction with VED from 73% at 22 J/mm^3^ up to 94.7% at 429 J/mm^3^. The increase of VED enhances the vaporization of austenite stabilizer elements, such as N, which causes the reduction of austenite content [26]. Moreover, Mulhi et al. [26] have also shown that the VED value influences the porosity of the 2507 alloy, as follows: (a) in the VED range of 22–68 J/mm^3^, porosity decreases from 46% to 5%; (b) in the VED range of 68–127 J/mm^3^, porosity decreases from 5% to 0.04%; and (c) in the VED range of 127–429 J/mm^3^, porosity increases from 0.04% to 1.56%. From such results, Mulhi et al. [26] identified the “process window” within which the alloy porosity due to lack of fusion, gas or metallurgical pore and the keyhole effect was minimized [27]. Davidson et al. [28] produced samples of the 2507 alloy by LPBF with VED in the range of 14–113 J/mm^3^. The resulting samples showed randomly distributed pores, which were attributed to both insufficient melting at lower laser powers and entrapped gases at higher energy densities. The highest reported sample density was 90.8% at VED = 71 J/mm^3^, with the as-built samples’ microstructure mainly formed of ferrite with small amounts of austenite preferentially located at the grain boundaries. Saeidi et al. [29] succeeded in manufacturing samples of the 2507 alloy with a ferrite volume fraction around 75%–78% and a relative density of 99.5%, using a VED value of 127 J/mm^3^. Kunz et al. [30] reported a relative density of 99.6% with a predominantly ferritic structure, using a VED value of 24 J/mm^3^.

A detailed analysis of closed and open porosity, respectively due to defects developed during the LPBF process and graded geometry [31], was not addressed in this study. However, based on the results experimentally obtained for S316L [15], it is expected that the open porosity of the SF53 scaffold is slightly lower than the nominal one from the STL file (P_STL_ = 72%). It is also noteworthy that size and distribution of the macro-pores influence the microstructure of the SF53 scaffold developed during the solidification process occurring after LPBF (Figure 4). The macro pores act as a thermal barrier causing poor heat dissipation, thus reducing the cooling rate at the pore–material interface.

VED-driven microstructure modification is the main effect responsible for the different mechanical behaviors of SF53 and S316L, both fabricated with dense-out geometry by LPBF, using the same manufacturing system (Figure 7a and Figure 7c, respectively). The 316L compressive response shows different deformation mechanisms of the graded structure simultaneously activated. The lack of oscillations observed in the compression curve (Figure 6) and the selective compaction of layers with thinner struts, as depicted in Figure 7d, indicate that throughout the deformation process, thinner struts enter the densification regime while thicker struts remain in the plastic yielding regime. This implies that the temporal progression of deformation in S316L experiences a time shift contingent upon the size of the strut.

The SF53 plastic regime, following the initial linear behavior, starts with the gradual collapse of the layer of elementary cells with the thinner strut thickness (0.25 mm). Thus, as for S316L, the predominant failure mechanism in SF53 initiates in correspondence with the thinner struts, due to high stress concentrations on strut junctions, as also reported by Onal et al. [32]. As the 0.25 mm struts of the three central elementary cells broke, due to the increasing compressive load, the elementary cells of the upper layer with a strut thickness of 0.50 mm interpenetrated into them (Figure 7b), while the two external cells of the 0.25 mm layer were flattened. This latter effect is responsible for the plastic plateau response reported in Figure 6. As the plateau trend ends, the densification of the collapsed material is responsible for the observed sharp compression increment, as for S316L (Figure 6). As a matter of fact, SF53 shows a mechanical ultimate compressive strength (σ_UC_) and elastic modulus that are respectively three times and up to seven times higher than S316L (Table 5), whose σ_UC_ value is very close to the experimental value of cortical bone (as reported in [15]).

Literature studies on 2705 DSS for bio-purposes mostly refer to corrosion behavior in simulated body fluids [33]. From an electrochemical viewpoint, cast DSS has better performance than 18Cr-12Ni-2Mo austenitic SS because of its lower susceptibility to localized corrosion in body-simulated media [3]. Furthermore, according to Cigada et al.’s [4] results in physiological solution, the 2507 DSS exhibits higher corrosion resistance in artificial saliva than the 2205 DSS (UNS S32205) [8]. The PREN of SF53 was considerably higher than that of the S316L, which ranges between 24 and 28 [15]. However, after up to 72 h of indirect biological tests, the medium conditioned with SF53 or S316L did not induce any cytotoxic effect on MG63 viability (Figure 8), proving the absence of the release of degradative products from both materials.

Several in vivo studies have been conducted on small animals aiming to understand the behavior of cast DSS in contact with biological tissues [6,13,34]. However, in this study, an in vitro assessment of 3D cell culture response was used in replacement of experiments requiring animal sacrifice, after Rai et al. [35]. The viability response of MG63 human osteoblast-like cells demonstrates that SF53 can be considered a good biocompatible material and a possible alternative to S316L for bone tissue engineering applications, allowing better viability as early as after seven days of culture (Figure 9A). Furthermore, it is worth noting that by increasing cell culture time from 24 h (Figure 9B,C) to seven days (Figure 9D,E), MG63 cells preferentially spread on powder particles partially melted on the as-built scaffold surface (Figure 3).

## 5. Conclusions

Duplex stainless steel (DSS) is emerging as an alternative to austenitic stainless steel (SS) for the manufacturing of temporary bone substitutes. The aim of this experimental work is providing some further insight with respect to the current know-how on the biomechanical performances of graded scaffolds based on the F53 alloy (UNS S32750 according to ASTM A182) produced by laser powder bed fusion (LPBF), for tissue engineering applications, using the in vitro behavior of 316L SS as a reference.

Following the results obtained in our previous work on the 316L SS scaffold (S316L), dense-out (DO) graded geometry based on the rhombic dodecahedral (RD) elementary unit cell was adopted to produce graded F53 DSS scaffolds (SF53) using the LPBF technology. The additive manufacturing process was carried out by using optimized parameters providing a volume energy density value Ev = 60 J/mm^3^. Microstructural characterization and mechanical and biological tests were performed on the SF53 and S316L scaffolds under similar conditions. The main results obtained in this study can be summarized as follows:

- The virgin new powder (PF53) with a particle size in the range of 5–50 μm and chemical composition compatible with the F53 alloy was mainly formed of α-Fe (ferrite) with about 6 wt.% of γ-Fe (austenite);
- The residual powder in the chamber after scaffold production (PF53R) showed larger particles sizes (5–130 µm), increased Mn content, and a lower amount of ferrite (α-Fe), with a larger crystallite size relative to PF53, due to the effect of thermal cycling occurring during the LPBF process;
- The SF53 scaffold is mainly formed of ferrite (80 wt.%) with smaller crystallites relative to the virgin new powder, due to the rapid cooling rates developed in the LPBF process;
- Compressive tests evidenced that plastic deformation in the graded lattices first involved the thinnest struts (0.25 mm) in both the SF53 and S316L scaffolds. However, contrary to S316L, SF53 showed a plastic regime followed by a plastic plateau, due to collapsing and breaking of the central elementary cells (0.25 mm thickness);
- The ultimate compressive strength (σ_UC_) and elastic moduli (E) of SF53 are three times and seven times higher than S316L, respectively;
- Biological assessment with MG63 human osteoblast-like cells demonstrated that the SF53 scaffold produced by LPBF with a dense-out geometry can be considered as a suitable biocompatible material and a viable alternative to 316L SS for bone tissue engineering applications, allowing better cell response already after seven days of culture.

## Figures and Tables

**Table 1:** Nominal composition (in wt.%) of the F53 raw powder from the manufacturer data sheet.

Fe	Cr	Ni	Mo	N	Mn	Si	Cu	P	C	S
Bal.	24–26	6–8	3–5	0.24–0.32	≤1.2	≤0.8	≤0.5	≤0.035	≤0.03	≤0.02

**Table 2:** Optimized printing parameters used to produce the SF53 scaffolds from the PF53 virgin new powder.

Parameter	Value
Laser power (W)	250
Scan speed (mm/s)	600
Hatching distance (mm)	0.14
Thickness (mm)	0.05

**Table 3:** Experimental chemical composition of samples obtained from EDS analysis. AV—average value; SD—standard deviation.

Element	Fe	Cr		Ni		Mo		Mn		Si	
		AV	SD	AV	SD	AV	SD	AV	SD	AV	SD
PF53	Bal.	25.4	0.3	7.4	0.4	3.4	0.2	1.0	0.1	0.43	0.05
PF53R	Bal.	25.8	0.2	7.3	0.2	3.2	0.1	1.5	0.2	0.7	0.2
SF53	Bal.	24.1	0.2	7.97	0.09	3.46	0.05	0.66	0.07	0.47	0.05

**Table 4:** Lattice parameter (a), relative amount (Quantity) and crystallite size (L) of α-Fe and γ-Fe phases estimated by peak shape analysis, Rietveld refinement and the Scherrer formula.

Sample	α-Fe			γ-Fe		
	a (nm)	Quantity (wt.%)	L (nm)	a (nm)	Quantity (wt.%)	L (nm)
PF53	0.2879 ± 0.0001	94 ± 1	28.6 ± 0.3	0.3624 ± 0.0001	6 ± 1	33 ± 5
PF53R	0.2877 ± 0.0001	80 ± 1	31.8 ± 0.1	0.3621 ± 0.0001	20 ± 1	31 ± 2
SF53	0.2881 ± 0.0001	80 ± 2	22.8 ± 0.3	0.3623 ± 0.0005	20 ± 2	24 ± 2

**Table 5:** Ultimate compressive strength ($\sigma_{UC}$) and elastic modulus (E) for SF35 and S316L graded lattice scaffolds with dense-out geometry. AV—average value; SD—standard deviation.

Scaffold	$\sigma_{UC}\;(\mathrm{MPa})$		E (GPa)	
	AV	SD	AV	SD
SF53	270	5	2.1	0.2
S316L	80	6	0.3	0.1

## Data Availability

Data sharing is not applicable to this article.
